# Assessment of linkages from HIV testing to enrolment and retention in HIV care in Central Mozambique

**DOI:** 10.7448/IAS.19.5.20846

**Published:** 2016-07-20

**Authors:** Celso Azarias Inguane, Stephen Gloyd, João Luis Manuel, Charlene Brown, Vincent Wong, Orvalho Augusto, Wisal Mustafa Hassan, Lúcia Vieira, Pires Afonso, Mehol Jamnadás, Jama Joy Bernard, James Cowan, Samuel Kalibala, James Pfeiffer

**Affiliations:** 1 Department of Anthropology, University of Washington, Seattle, WA, USA; 2 Health Alliance International, Seattle, WA, USA; 3 Department of Global Health, University of Washington, Seattle, WA, USA; 4 Centro de Investigação Operacional de Beira (CIOB), Ministry of Health, Beira, Mozambique; 5 Office of HIV/AIDS, USA, Agency for International Development, Washington, DC, USA; 6 Faculdade de Medicina, Universidade Eduardo Mondlane, Maputo, Mozambique; 7 Manhiça Research Center (CISM), Manhiça, Mozambique; 8 HIVCore/Population Council, Washington, DC, USA

**Keywords:** linkages, retention, HIV care, treatment, Mozambique

## Abstract

**Introduction:**

Effectiveness of the rapid expansion of antiretroviral therapy (ART) throughout sub-Saharan Africa is highly dependent on adequate enrolment and retention in HIV care. However, the measurement of both has been challenging in these settings. This study aimed to assess enrolment and retention in HIV care (pre-ART and ART) among HIV-positive adults in Central Mozambique, including identification of barriers and facilitators.

**Methods:**

We assessed linkages to and retention in HIV care using a mixed quantitative and qualitative approach in six districts of Manica and Sofala provinces. We analyzed routine district and health facility monthly reports and HIV care registries from April 2012 to March 2013 and used single imputation and trimmed means to adjust for missing values. In eight health facilities in the same districts and period, we assessed retention in HIV care among 795 randomly selected adult patient charts (15 years and older). We also conducted 25 focus group discussions and 53 in-depth interviews with HIV-positive adults, healthcare providers and community members to identify facilitators and barriers to enrolment and retention in HIV care.

**Results:**

Overall, 46% of the monthly HIV testing reports expected at the district level were missing, compared to 6.4% of the pre-ART registry reports. After adjustment for missing values, we estimated that the aggregate numbers of adults registered in pre-ART was 75% of the number of persons tested HIV-positive in the six districts. In the eight health facilities, 40% of the patient charts for adults enrolled in pre-ART and 44% in ART were missing. Of those on ART for whom charts were found, retention in treatment within 90 and 60 days prior to the study team visit was 34 and 25%, respectively. Combining these multiple data sources, the overall estimated retention was 18% in our sample. Individual-level factors were perceived to be key influences to enrolment in HIV care, while health facility and structural-level factors were perceived to be key influences of retention.

**Conclusions:**

Efforts to increase linkages to and retention in HIV care should address individual, health facility, and structural-level factors in Central Mozambique. However, their outcomes cannot be reliably assessed without improving the quality of routine health information systems.

## Introduction

In the past decade, Mozambique has expanded access to HIV testing services and antiretroviral therapy (ART) and has integrated them into the primary healthcare system [1–4]. Still, the proportion of people with an HIV-positive diagnosis who have been linked to and retained in HIV care has been reported to be low [5,6], similar to much of sub-Saharan Africa [7,8]. The Mozambique Ministry of Health reported in 2015 that ART coverage was only 59% of the estimated treatment needs [9], and, as in other countries in the region, the proportion of those retained in pre-ART care and ART in Mozambique tends to decrease over time [8–10]. This poses challenges to reaching the ambitious 90-90-90 goals of the Joint United Nations Programme on HIV/AIDS’ (UNAIDS) [11,12]. The new WHO guidelines on early initiation of ART [13] may contribute to achieving those goals, but only if people who are diagnosed HIV-positive are enrolled and retained in care. Moreover, accurate monitoring of enrolment and retention with reliable information about the HIV cascade [14] is critical for making progress in HIV care. Nevertheless, poor HIV data quality from both routine sources and international implementing partners [9,15] remains a challenge to understanding the cascade.

A study aimed to identify and measure loss to follow-up in the HIV care cascade in Manica and Sofala provinces, Central Mozambique, using adjusted routine health systems data and patient charts to estimate the proportion of people with an HIV-positive diagnosis who were linked to and retained in pre-ART care and ART. The study also aimed to identify perceived barriers and facilitators of enrolment and retention in pre-ART care and ART.

## Methods

### Study setting and design

We conducted this mixed methods study in six districts: Beira, Buzi and Dondo in Sofala Province; and Barue, Chimoio and Manica in Manica Province. Beira and Chimoio are the capital cities of Sofala and Manica provinces, respectively. The other four districts are rural. We selected these six districts because of their rural-urban mix, their mix of different levels of health services, and their security. These districts are mostly contiguous and cover most of the major health facilities in Central Mozambique. Self-referral to health facilities inside or outside this six-district region would likely have been infrequent. At the time of the study, HIV testing, pre-ART care and ART were free and widely available throughout the country.

To obtain an overview of HIV testing and HIV care enrolment at the district level, we examined routine monthly reports from all (87) health facilities that provided HIV testing or treatment in those districts. To obtain an in-depth assessment of enrolment, patient flow and retention in pre-ART care and ART at the health facility level, we selected eight of the 87 facilities for further study. The eight facilities were among the 34 facilities that provided ART during the study period; four were the main referral health facilities in rural districts and four were moderate to large patient volume facilities in urban areas.

#### District overview assessment

We collected and analyzed routine district and health facility monthly reports of HIV testing and HIV testing registries (pre-ART, ART) in each of the six districts from April 2012 to March 2013 (the study period). The study start date corresponds with a major revision of HIV data collection and monitoring registries, including new patient forms, registry books and reporting templates, that had been introduced in Mozambique in early 2012. We also collected data directly from HIV registries at district-level health offices, from pharmacy records of tests distributed and reported positive and from the national electronic health information system, locally known as the *Módulo Básico*.

We conducted descriptive analyses such as frequency for HIV tests, pre-ART and ART registrants, and for missing values in the routine data set. We examined missing value patterns over time and by health facility, and tested several imputation methods to substitute missing values: (1) single imputation using mean, trimmed means (replacing values above 50% of the mean for facilities that had less than five months data) and median, (2) Poisson generalized linear modelling and (3) iterative singular value decomposition (SVD) method (specifically rank-1 SVD approximation). None of these methods yielded consistently higher or lower imputed values than the other methods. We finally used the single imputation method because it gave the most conservative values. We performed the imputations in R (version 3.2) and analysis in Stata 13.1.

#### Health facility quantitative assessment

At each of the eight health facilities, we abstracted enrolment data from the pre-ART and ART registry books and retention data from patient charts and pharmacy records. Mozambique Ministry of Health policy stipulates that (1) all newly diagnosed HIV-positive patients be first registered in pre-ART registry books (even those who are eligible for ART) and (2) patient charts be created for all pre-ART and ART patients. The pre-ART and ART registry books included dates of HIV-positive and CD4 tests and the dates of ART initiation. We conducted a stratified random sample of registrants from the pre-ART and ART registries separately (using random.org) to obtain 100 charts of patients older than 15 years from each facility, excluding women in prevention of mother-to-child transmission of HIV (PMTCT) programmes. We searched for the selected patient charts in archives, consultation rooms and other parts of the health facilities. Data abstracted from patient charts included demographic characteristics, site of testing, and dates for ordering CD4 tests, receipt of CD4 results, enrolment into care, ART initiation, consultations, ART pickups, and the date of the study team visit. Data were entered into study computers using EPIDATA 3.2 and exported for cleaning and analysis in Stata 13.1.

To construct the overall HIV cascade, we used different data sources for enrolment and retention. Enrolment proportions were measured in two steps: first, by the number of people, district-wide, registered in pre-ART divided by the estimated (adjusted) number of newly diagnosed HIV positive in each district during the study period; and second, by the proportion of charts found among the selected people registered on pre-ART and ART at each of the eight health facilities. Retention rates were measured by the proportion of patient charts with evidence of an ART clinic visit or antiretroviral (ARV) pickup by patient charts or individual pharmacy records within 30, 60 or 90 days prior to the study team visit to the facility. Retention was examined separately for pre-ART and ART patients.

#### Health facility qualitative assessment

At each of the eight facilities and their surrounding areas, we conducted in-depth interviews (IDIs) with health facility directors and with people living with HIV who (1) enrolled in pre-ART care within 30 days of their HIV-positive test result, (2) enrolled after 30 days from their HIV-positive test result, (3) enrolled but eventually dropped out of pre-ART care and (4) never enrolled in HIV care. In each facility, we had aimed to include at least one person living with HIV from each of the four categories, and adolescents (18–19 years old). Participants were at least 18 years old. Study interviewers contacted and obtained informed consent directly from facility directors before conducting an IDI. During health facility visits, healthcare providers informed people living with HIV eligible for study participation about the study and gave them the option of contacting study interviewers for informed consent if they were interested in participating in the study. In health facility surrounding areas, facility-based outreach workers provided information about the study to people who had dropped out of care or who had never enrolled in care, and gave them the option of contacting study interviewers for informed consent if they were interested in participating in the study. Study interviewers also conducted focus group discussions (FGDs) with healthcare providers, community outreach workers, and members of patient-support groups at the health facility. They conducted FGDs with community leaders in community meeting rooms. IDIs and FGDs focused on people's experience with HIV testing and HIV care, barriers and facilitators of enrolment and retention in pre-ART care and ART, and on visualizing those experiences, barriers and facilitators through developing patient flow maps in each facility.

Study interviewers took notes and audio-recorded IDIs and FGDs to expand notes and improve reliability. We coded and analyzed notes using ATLAS.ti 7^TM^ (www.atlasti.com/). This paper reports only on the main facilitators and barriers for linkages and retention in pre-ART care and ART, that is, those mentioned by at least three out of the four types of people living with HIV, and in at least four out of the eight study sites. More in-depth analysis of the qualitative data is planned for future publication.

#### Ethical considerations

The Institutional Review Boards of the Population Council, United States, and the Mozambique National Institute of Health (CIBS-INS) approved the study. All participants provided written informed consent. In the published figures and tables, we replaced actual names of health facilities with anonymous alphabetical codes to avoid adverse events that might result from reporting on low performance.

## Results

At the district level, we analyzed all available reports from the 87 facilities providing HIV testing and the 34 facilities providing ART. At the eight health facilities providing ART, we abstracted information from 795 patient charts (430 pre-ART and 365 ART) and we conducted 25 FGDs (comprising 248 participants) and 53 IDIs. We excluded three low-quality IDIs from analysis, leaving 23 IDIs with people who enrolled within 30 days of being diagnosed HIV-positive, seven with people who had enrolled 30 days or more after their HIV-positive test result, 10 with people who had enrolled in care and dropped out and two with HIV-positive people who never enrolled in care. We also conducted eight IDIs with health facility directors.

### District-level HIV testing and enrolment in care

Monthly reports submitted at the district level indicated that slightly more people tested HIV-positive than the number of people newly registered in pre-ART care (Table 1). Beira and Manica districts reported fewer people who tested HIV-positive than those registered in pre-ART. Overall, 46% (range 25–65% among districts) of the 1944 monthly reports for HIV testing that were expected from the 87 facilities were missing, compared to 6.4% (range 2–15%) of the 312 expected monthly reports for registry in pre-ART and ART. Missing values were more frequent from the electronic health information system, including three districts without reports for any HIV testing data during the study period. After imputation adjustment, the proportional increase from raw totals was 35% for people tested HIV-positive and only 4.2% for those registered in pre-ART care. With the adjustment, the overall proportion of HIV-positive people who enrolled in pre-ART care in the six districts was 75% (range 46–92%).

**Table 1:** Monthly reported and adjusted numbers and percentages of people with HIV-positive diagnosis and enrolled in pre-ART between April 2012 and March 2013 in six districts in Manica and Sofala provinces

	Number of people with HIV-positive diagnosis		Number of people with HIV-positive diagnosis enrolled in pre-ART		Proportion of people with HIV-positive diagnosis enrolled in pre-ART	
			
Districts	Reported	Adjusted	Reported	Adjusted	Reported (%)	Adjusted (%)
Búzi	2104	3363	1549	1658	74	46
Dondo	2898	2757	1681	2681	58	56
Beira	8143	12,040	11,061	11,061	136	92
Manica	2404	3498	2660	2660	111	76
Bárue	2096	2164	1194	1194	57	55
Chimoio	7032	7406	5675	5675	81	77
Total	24,677	33,228	23,820	24,820	97	75

Note: Adjusted numbers combined reported values for reported months and imputed values for missing months.

#### Enrolment in HIV care in the eight ART facilities

The overall mean proportion of pre-ART and ART registrants whose charts were located at the health facilities was 60 and 66%, respectively, with a high variation between the facilities (range 31–85% for ART and 7–64% for pre-ART). Patient demographics abstracted from the charts were not significantly different between pre-ART and ART patients (Supplementary Table 1). Pre-ART and ART samples from the eight health facilities had similarly high proportions of women (62% pre-ART and 55% ART). Patients were mostly between 15 and 49 years old (91% pre-ART and 92% ART), and most had primary and secondary level of education (68% pre-ART and 69% ART). The socio-demographic profiles of HIV-positive persons interviewed were similar to those whose charts were reviewed: 95% were between 20 and 49 years of age, 61% were women, 57% reported completing primary and 31% reported completing secondary or higher education level. Thirty-eight percent of interviewees reported being married or in marital union, 21% widowed and 77% reported having children. Regarding occupations, 33% reported being domestic workers, 24% small business owners and 15% workers in agriculture/fishing.

**Figure 1:**
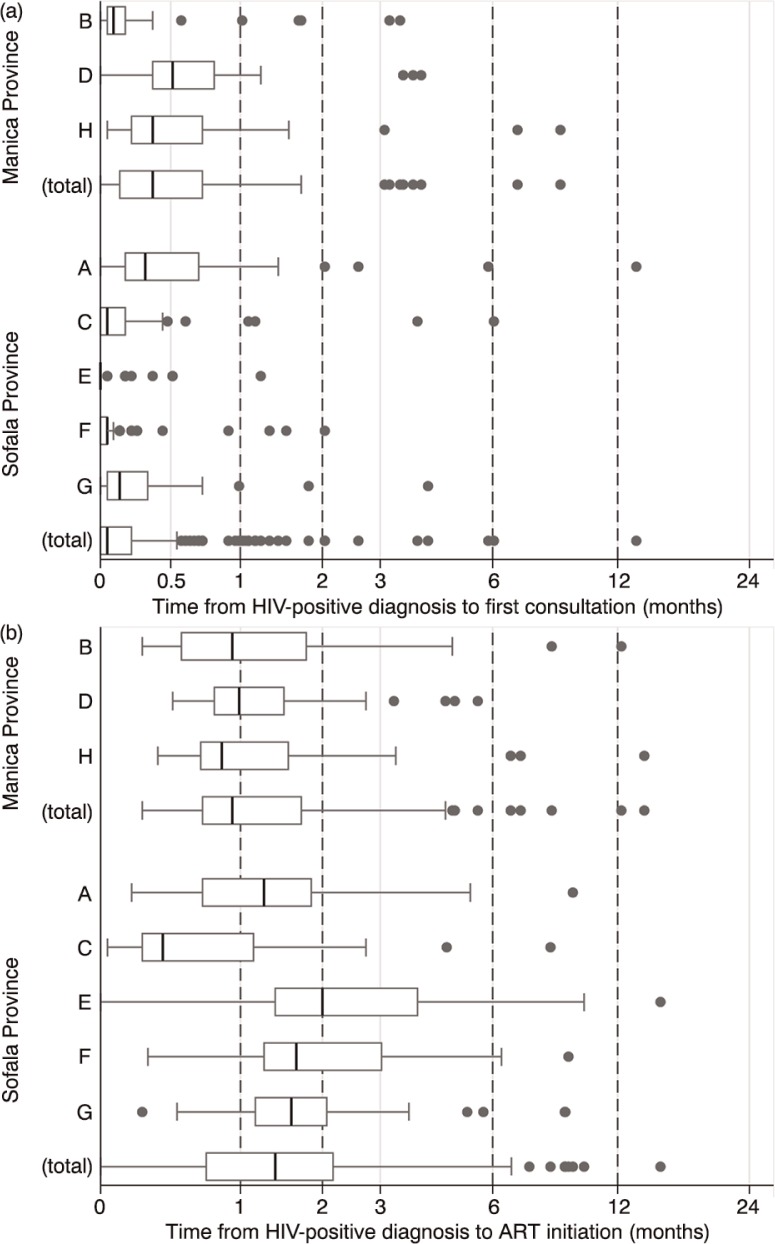
Months from HIV-positive diagnosis to first consultation among pre-ART patients (a), and time from HIV-positive diagnosis to ART initiation among ART patients (b), in eight health facilities in Manica and Sofala provinces.

Patient chart data demonstrated a large variation between the health facilities in time from HIV-positive test to first clinical consultation (overall mean 13.3 days; IQR 0.0–12.0 days; range 0.0–13.3 months) and in time from HIV diagnosis to ART initiation (overall mean 1.8 months; IQR 0.7–2.1 months; range 0.1–12.2 months) (Figure 1).

#### Retention in ART in the eight ART facilities

**Figure 2:**
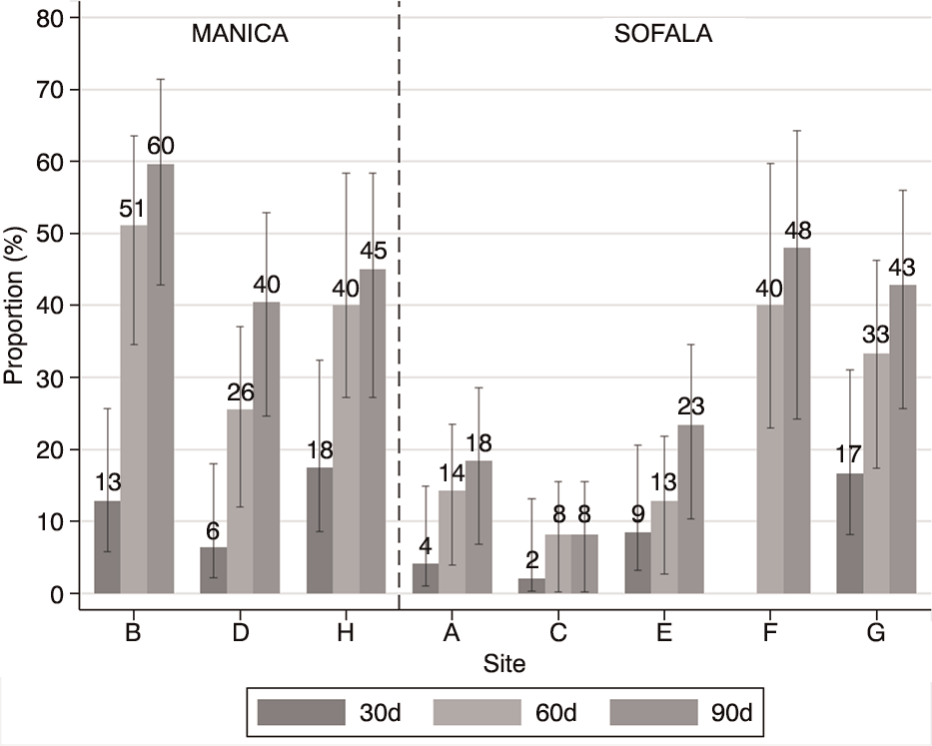
Proportion of ART patient charts with evidence of clinical visit or ARV pickup, 30, 60 and 90 days before study visit in eight health facilities of Manica and Sofala provinces.

Among the 346 ART charts found, the median time from the initiation of treatment until study team visit was 18.6 months. Overall, 34, 27 and 8% of these ART charts had evidence of a clinic visit or ARV pickup within 90, 60 or 30 days of the study team visit, with a wide variation among the eight health facilities (Figure 2). We found that the number of days for which ARVs were dispensed was frequently missing. However, during IDIs and FGDs, health providers consistently reported that ARVs were usually dispensed for 60 days or occasionally for 90 days when patients were clinically stable.

Crude and adjusted logistic regression did not show differences in retention rates between males and females. However, retention (evidence of ARV pickup within 90 days of study visit) in Manica Province was significantly greater than in Sofala Province (Supplementary Table 2).

#### Overall HIV care cascade

**Figure 3:**
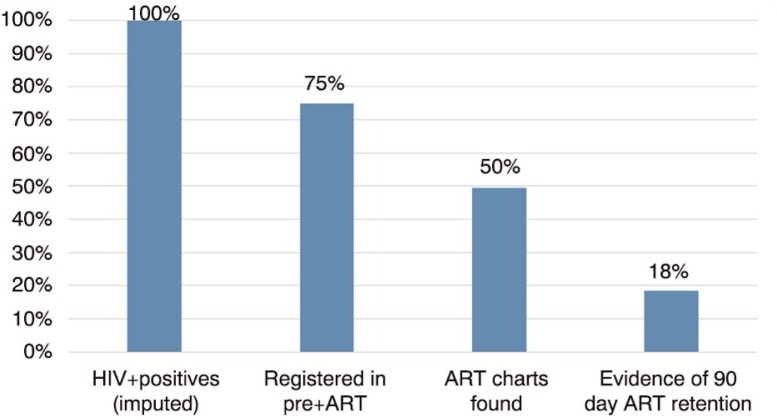
Overall HIV cascade – from HIV testing to enrolment to charts located to retention – aggregate data from eight health facilities in Manica and Sofala provinces. Note: Each bar represents the proportion of the original total of people tested HIV-positive, for example, 50% of HIV-positive charts found=66% charts found times 75% registered. Adjusted numbers combined reported values with imputed values for missing months.

We constructed an overall HIV care cascade (Figure 3) using the following sources of data: (1) district-level proportions of estimated HIV-positive people who were registered in pre-ART, (2) proportions of patients registered in ART in the eight health facilities whose charts were found and (3) proportions of those patient charts with any evidence of clinic visit or ARV pick up within 90 days of our study team review visit. The calculations suggest that, in aggregate, 18% of the HIV-positive patients diagnosed in health facilities during the study period were retained in treatment one to two years later.

### Perceived facilitators and barriers to HIV testing, enrolment and retention

Most respondents from both the IDIs and FGDs said that they preferred to be tested at sites that provided ART and near their places of residence. Both men and women who enrolled early in HIV care said that the main reason for obtaining an HIV test – and for enrolling in HIV care – was the presence of symptoms of sickness. Conversely, the main perceived barrier for enrolment was lack of symptoms of sickness. Some women who enrolled early mentioned their desire to protect their children from vertical transmission and wishing to live longer to raise their children.

Only early enrollers in HIV care mentioned a facilitator of retention, living near the health facility where one receives HIV care. Perceived barriers for retention included: (1) the perception of poor and disrespectful service at the health facility, especially for people on ART who were late to their ARV pickup appointments and (2) lack of money for food and for transportation.

Table 2 summarizes facilitators and barriers to enrolment and retention in HIV care that were mentioned during IDIs and FGDs.

**Table 2:** Perceived facilitators and barriers to enrolment and retention in HIV care in eight health facilities in Manic and Sofala provinces

	Facilitators	Mentioned by	Barriers	Mentioned by
**Enrolment in HIV care**	Presence of symptoms	Early enrollers Community leaders	Lack of symptoms	Late enrolment, never enrolled, dropouts, patient-support group and healthcare providers
**Retention in HIV care**	Proximity to health facility	Early enrollers	Disrespect by health workers, poor quality of healthcare Lack of money for food and transport	Early enrolment, late enrolment, dropouts, patient-support group, healthcare providers and outreach workers Early enrolment, late enrolment and dropouts

Healthcare providers described how HIV-positive patients could be lost at different steps along the HIV continuum of care through a variety of delays and loss to follow-up in patient flow that varied by site. For example, losses were especially common in the intervals between patients receiving their HIV-positive result and creating the chart, or between having their blood drawn for laboratory tests and receiving those results. Interviewees also mentioned that, at all eight health facilities, charts were not created in the same room where HIV tests were done and results disclosed. In some health facilities, patients were sent to queue at the health facility reception to have their charts created when frequently charts were not created at all. At other facilities, providers said they took the patient's test results and went to create the chart themselves at the reception. When blood samples were sent to other health facilities to obtain CD4 counts, the results often took several weeks to be returned to patients.

**Figure 4:**
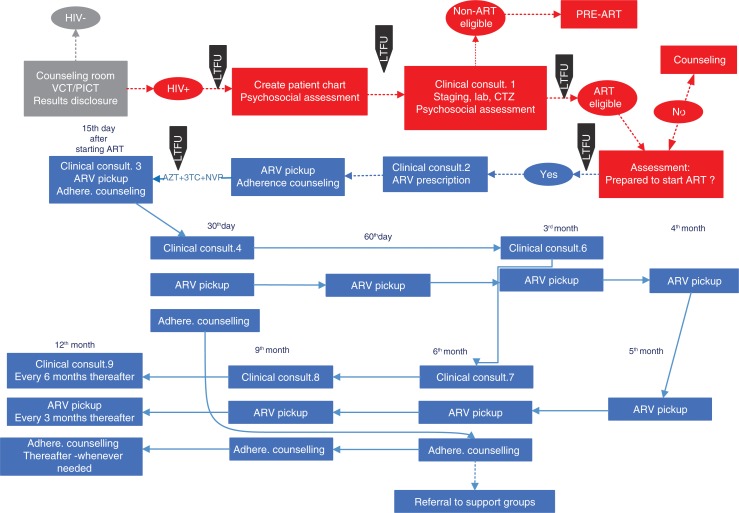
Patient flow map showing loss to follow up along the HIV continuum of care in eight health facilities of Manica and Sofala provinces. ART: antiretroviral therapy; AZT+3TC+NVP: zidovudine+stavudine+niverapine; CTZ: cotrimoxazole; HIV+: HIV-positive; PICT: provider initiated counselling and testing; LTFU: lost to follow-up; VCT: voluntary counselling and testing.

We constructed Figure 4 that shows an idealized flow map for HIV-positive patients in ART, based on the Mozambique 2014 national ART guidelines. The information gathered from our eight FGDs with 74 healthcare providers at the eight study health facilities suggested specific points in the flow map where patients were typically lost to follow-up (LTFU), noted with black downward-pointing arrows with an LTFU label.

## Discussion

This study demonstrated substantial losses to follow-up of HIV-positive persons at all points along the HIV care and treatment cascade in Central Mozambique. Moreover, our findings suggest that the routine HIV information, as reported, grossly overestimates both the true enrolment and retention in HIV care. Our findings also suggest that HIV-positive patients experienced major facilitators and barriers for enrolment and retention in HIV care, including individual, health system and structural factors.

Our estimate of the proportion of people diagnosed with HIV who are registered in pre-ART care was slightly higher than other estimates in sub-Saharan Africa [8]. Our overall retention estimates are much lower than nationally reported retention estimates in Mozambique for 2014 (67% at 12 months after ART initiation, and 56% at 24 months) [9,12]. Our low retention estimates are, however, not substantially different than other carefully conducted studies in sub-Saharan Africa [7,11,12]. Our facilitators for enrolment in pre-ART and ART care (e.g. proximity to ART services and severity of illness) were similar to studies elsewhere [16,17]. Yet, in contrast to those studies, facilitators for enrolment and retention in our study seemed to only help those who enrolled early in care (within 30 days of their HIV diagnosis). Our barriers for retention (e.g., lack of symptoms, perceived poor quality of healthcare) were consistent with other studies in sub-Saharan Africa [16–18].

The large variations in patient flow patterns, in observance of national norms, and LTFU found among the eight ART facilities suggest that targeted health systems change, including simplification of patient flow, improved patient information and improving health worker behaviour, might substantially improve performance. The data also suggest that there is an urgent need for strategies to re-link patients into HIV care in Mozambique, using lessons learned from the country and other low-resource settings [19]. Strategies might include having healthcare workers, with whom many patients have developed close relationships, contact patients who have dropped out and understand reasons why they dropped out [19]. Judgemental attitudes or threatening patients is not helpful, as has been reported in other sub-Saharan African contexts [20]. However, in Mozambique, many of these healthcare workers battle with work overload [21,22] or lack of motivation because they feel “exploited and ultimately abandoned” by the nature of global HIV interventions [23]. Working conditions need to be addressed to complement training. Improving workforce morale, understanding individual patient circumstances (including reasons beyond their control) and bearing in mind that missing visits are inevitable over the lifelong course of HIV care can help in the process of re-linking patients into care [20]. Another strategy, based on our results, would be to simplify algorithms of patient care and confusing patient flow patterns, and to address challenges with patient chart management.

Our finding that a large proportion of those who register in pre-ART (who are thus considered “linked” to HIV care) have no evidence of subsequent encounters with the health system is disturbing. The finding raises serious concerns about using registration at ART facilities as an indicator for effective enrolment. Measuring retention is also a challenge. If retention is measured only among those with patient charts or pharmacy records, the results will overestimate the true retention of the people originally tested HIV-positive in the health system. On the other hand, incomplete recording of visits or ARV pickup in patient charts may underestimate true retention of those in care with charts. Follow-up of pharmacy records can complement data on patient retention if the pharmacy records are adequately filled out. All enrolment and retention estimates, however, will be meaningful only when they are based on an accurate denominator of the total numbers of people tested HIV-positive in the health system.

The Mozambique Ministry of Health has acknowledged poor HIV data quality nationally [9,15] and data concerns have been well documented through ethnographic research in Mozambique [21]. Authors concerned about data quality in other countries have called for the use of multiple measures of linkages to HIV care and retention in HIV care and treatment [24–26]. Other lower resolution proxy measures (e.g. viral load) may provide broader generalizability for the measurement of trends in linkage to care over time [27]. We hope that our research will contribute to a deeper understanding of how to use proxy measures of linkage to HIV care and treatment to match the desired programmatic or clinical outcomes.

## Limitations

The poor quality of reported data was both a principal finding of the study and a limitation. Although our imputation methods were designed to supplement routine data to obtain more accurate estimates, we cannot be certain that the adjusted estimates reflect the true picture of registry and enrolment in care. Patients may have received effective ART without adequate evidence of their treatment in patient charts. We noted earlier that the data systems in Mozambique did not allow for analysis of individual linkages of people tested HIV-positive to HIV care, since no codes were used for testing, and as a result testing could not be linked to registries in the ART sites. Thus, it was not possible to ascertain the extent to which people tested HIV-positive might be obtaining care from elsewhere, including outside the study districts. Since transfers of patients were rarely registered, our estimates of retention likely underestimated the true retention of our sample HIV-positive registrants.

The qualitative assessment was limited by the small numbers of HIV-positive people who enrolled late, dropped out or never enrolled in care that we were able to interview. Moreover, the findings related to health systems barriers can only represent the practices at these eight facilities. Other facilities might have presented substantially different qualitative (and quantitative) findings. Nevertheless, our diverse sample that included perspectives of people living with HIV, health providers, and community outreach workers and members provided wide-ranging perceptions.

## Conclusions

Our findings suggest that these districts in Central Mozambique face serious challenges to effectively enrol and retain people who are tested HIV-positive in the health system. The poor quality of routine reported data, especially regarding HIV testing, is a major barrier to identifying these bottlenecks. More attention needs to be focused on improving the quality and analysis of routine data regarding all steps of the HIV cascade at each health facility. We think that our careful utilization of routine health system information, which included data quality assessment, triangulation of data from other sources, adjustment by imputation for missing data, and patient chart review, provided a reasonably high-resolution measure of linkage to care. The HIV care cascade model that we constructed provided health facility-specific detail on critical bottlenecks from which tailored interventions can be developed. Our study results also suggest that health system, individual and structural factors were important perceived barriers to enrolment and retention in care. Modifying these factors and assessing their impact with reliable data should substantially improve linkages to enrolment and retention in HIV care and enhance global efforts to address HIV.

## Supplementary Material

Assessment of linkages from HIV testing to enrolment and retention in HIV care in Central Mozambique
