# Supplementary material for: Assessment of linkages from HIV testing to enrolment and retention in HIV care in Central Mozambique
Source: J Int AIDS Soc. 2016 Jul 20;19(5Suppl 4):20846. doi: 10.7448/IAS.19.5.20846 (PMC4956731; doi:10.7448/IAS.19.5.20846)
Supplement: Assessment of linkages from HIV testing to enrolment and retention in HIV care in Central Mozambique [file JIAS-19-20846-s001.pdf]

Supplemental Table 1. Demographic characteristics of the pre-ART and ART charts included in eight health facilities of Manica and Sofala Provinces

	Pre-ART		ART	
**Characteristic**	**N**	**%**	**N**	**%**
Total	430		365	
Male	165	38.4	165	45.2
Age, mean (SD)	32.9 (10.52)		34.2 (9.93)	
15 - 24	98	22.8	55	15.1
25 - 34	167	38.8	152	41.6
35 - 49	128	29.8	129	35.3
50 - +	37	8.6	29	7.9
Education				
No schooling	72	16.7	45	12.3
Primary	178	41.4	143	39.2
Secondary or more	113	26.3	110	30.1
Miss	67	15.6	67	18.4
Site Province				
Manica	157	36.5	138	37.8
Sofala	273	63.5	227	62.2

Supplemental Table 2. Evidence of clinical visit or ARV pickup, 30, 60 and 90 days before study visit for ART patients in eight health facilities of Manica and Sofala Provinces

		Unadjusted*		Adjusted**†	
	**N**	**Prop**	**95% CI**	**Prop**	**95% CI**
All sites					
Total charts reviewed	346	-	-	-	-
in last 30 days	30	8.7	5.7 - 11.6	8.0	4.2 - 11.9
in last 60 days	93	26.9	22.1 - 31.4	26.0	16.9 - 34.0
in last 90 days	119	34.4	29.2 - 39.2	33.7	22.7 - 43.1
Manica Province sites					
Total charts reviewed	134	-	-	-	-
in last 30 days	16	11.9	6.5 - 17.4	11.4	5.4 - 17.5
in last 60 days	52	38.8	30.0 - 46.5	38.2	26.1 - 48.4
in last 90 days	65	48.5	39.3 - 56.3	48.2	34.5 - 59.0
Sofala Province sites					
Total charts reviewed	212	-	-	-	-
in last 30 days	14	6.6	3.2 - 9.9	6.3	2.0 - 10.6
in last 60 days	41	19.3	13.8 - 24.5	19.0	9.2 - 27.7
in last 90 days	54	25.5	19.4 - 31.1	25.3	13.0 - 35.8
Female					
Total charts reviewed	199	-	-	-	-
in last 30 days	21	10.6	6.3 - 14.8	10.0	5.6 - 14.5
in last 60 days	58	29.1	22.5 - 35.2	28.5	20.6 - 35.6
in last 90 days	78	39.2	32.0 - 45.6	39.1	29.8 - 47.1
Male					
Total charts reviewed	147	-	-	-	-
in last 30 days	9	6.1	2.2 - 10.0	5.7	1.6 - 9.8
in last 60 days	35	23.8	16.6 - 30.4	23.2	13.4 - 31.9
in last 90 days	41	27.9	20.3 - 34.8	27.3	16.4 - 36.9

CI - confidence interval

* 95% CI computed through discrete time logistic regression

** Adjusted for age and sex

† Estimates from a random-intercepts logistic regression to address clustering per site

Supplemental Table 3. Evidence of clinical visit or ARV pickup, 30, 60 and 90 days before study visit per study site for ART patients in eight health facilities of Manica and Sofala Provinces

		In last 30 days			In last 60 days			In last 90 days		
	**Total**	**N**	**Prop**	**95% CI**	**N**	**Prop**	**95% CI**	**N**	**Prop**	**95% CI**
All sites	346	30	8.7	5.7 - 11.6	93	26.9	22.1 - 31.4	119	34.4	29.2 - 39.2
Site A	49	2	4.1	1.0 - 14.9	7	14.3	3.9 - 23.5	9	18.4	6.8 - 28.5
Site B	47	6	12.8	5.8 - 25.6	24	51.1	34.5 - 63.5	28	59.6	42.8 - 71.4
Site C	49	1	2.0	0.3 - 13.1	4	8.2	0.2 - 15.5	4	8.2	0.2 - 15.5
Site D	47	3	6.4	2.1 - 18.0	12	25.5	12.0 - 37.0	19	40.4	24.6 - 52.9
Site E	47	4	8.5	3.2 - 20.6	6	12.8	2.7 - 21.8	11	23.4	10.3 - 34.6
Site F	25	0	-	-	10	40.0	23.0 - 59.7	12	48.0	24.2 - 64.3
Site G	42	7	16.7	8.2 - 31.0	14	33.3	17.4 - 46.2	18	42.9	25.7 - 56.0
Site H	40	7	17.5	8.6 - 32.4	16	40.0	27.2 - 58.4	18	45.0	27.2 - 58.4
